# Comprehensive analysis of lncRNAs and mRNAs in skeletal muscle of rainbow trout (*Oncorhynchus mykiss*) exposed to estradiol

**DOI:** 10.1038/s41598-017-12136-6

**Published:** 2017-09-18

**Authors:** Jian Wang, Prasanthi P. Koganti, Jianbo Yao, Shuo Wei, Beth Cleveland

**Affiliations:** 1 Division of Animal and Nutritional Sciences, West Virginia University, Morgantown, WV 26506 USA; 2 USDA/ARS National Center for Cool and Cold Water Aquaculture, Kearneysville, WV 25430 USA; 3 Department of Computational Biology, St. Jude Children’s Research Hospital, Memphis, TN 38105 USA; 4 Department of Biological Sciences, University of Delaware, Newark, DE 19716 USA

## Abstract

Estradiol (E2) is a steroid hormone that negatively affects muscle growth in rainbow trout (*Oncorhynchus mykiss*), but the mechanisms directing with this response are not fully understood. To better characterize the effects of E2 in muscle, we identified differentially regulated mRNAs and lncRNAs in juvenile rainbow trout exposed to E2. Here, we performed next-generation RNA sequencing and comprehensive bioinformatics analyses to characterize the transcriptome profiles, including mRNAs and long noncoding RNAs (lncRNAs), in skeletal muscle of rainbow trout injected with E2. A total of 226 lncRNAs and 253 mRNAs were identified as differentially regulated. We identified crucial pathways, including several signal transduction pathways, hormone response, oxidative response and protein, carbon and fatty acid metabolism pathways. Subsequently, a functional lncRNA-mRNA co-expression network was constructed, which consisted of 681 co-expression relationships between 164 lncRNAs and 201 mRNAs. Moreover, a lncRNA-pathway network was constructed. A total of 65 key lncRNAs were identified that regulate 20 significantly enriched pathways. Overall, our analysis provides insights into mRNA and lncRNA networks in rainbow trout skeletal muscle and their regulation by E2 while understanding the molecular mechanism of lncRNAs.

## Introduction

The recent advent of next generation RNA sequencing (RNA-Seq) and publication of reference genomes for many organisms have allowed researchers to study transcription profiles. The majority of the mammalian genome (up to 80%) is transcribed, of which only 2–3% are protein-coding RNAs (mRNAs) and the rest are noncoding RNAs. Most transcribed ncRNAs are larger than 200 nucleotides and defined as long non-coding RNAs (lncRNA) with some further processed to generate small RNAs^1^. LncRNA have fewer, longer exons when compared to coding genes and exhibit cell-type specific expression. These long non-coding RNAs are reported to play critical roles in various biological processes, including chromatin modification, regulation of transcription, influence of nuclear architecture and regulation of gene expression at post-transcriptional and post-translational levels, and are also reported to interact with DNA, RNA and proteins during these processes^2^. Availability of sequencing techniques led to the discovery of non-coding transcripts involved in regulation of gene expression, as well as novel protein coding genes.

Studies in humans (*Homo sapiens*) and mice (*Mus musculus*) support that lncRNA are involved in transcriptional regulation of skeletal muscle development and differentiation^2^. For instance, ChiP-Seq studies identified two different lncRNAs (^DRR^RNA and ^CE^RNA) that transcribe from the enhancer region of myogenic transcription factor MyoD^2,3^ to increase expression of MyoD and myogenin. An additional study identified a number of muscle specific lncRNAs regulated by Yin Yang 1 protein that function as both positive or negative regulators of muscle differentiation^4^. Expanding the investigation of lncRNAs as myogenic regulators in additional species is critical to both 1) establish whether previously reported mechanisms are conserved and 2) identify novel mechanisms within a species.

Rainbow trout (*Oncorhynchus mykiss*) is a fish species valued as a research model, food source, and recreational fish. This species demonstrates a pattern of indeterminate growth, or the ability to grow in length and continually accumulate muscle throughout its lifespan^5^. Muscle regulatory factors (MRF) such as MyoD and myogenin that regulate hyperplasia and hypertrophy in mammalian muscle are also important for these processes in salmonids^6^. However, unique MRF expression patterns occur during differentiation of rainbow trout myogenic precursor cells (MPCs)^7^, suggesting that these mechanisms are significant for continued hyperplasic capacity and indeterminate growth.

Determining how lncRNAs are regulated by biological factors that affect muscle growth is central for identifying lncRNA-related mechanisms important for myogenesis. In salmonids, estradiol (E2) is a biological factor that negatively effects muscle growth^8,9^, partially through direct effects on MPC proliferation^10,11^ and protein turnover^12,13^. Plasma E2 levels increase during sexual maturation and its catabolic effects in muscle are important for directing nutrients away from muscle growth and toward gonad development^8,14^, an energy intensive process that compromises muscle quality^15,16^. Our previous findings support a role for noncoding RNAs in the short-term E2-response (24 hr post-injection), specifically through regulation of miRNAs that directly affect expression of genes related to proteolysis, cell differentiation, and cell proliferation^11^. The present study aims to identify the differentially expressed lncRNAs and mRNAs in skeletal muscle exposed to E2 and create a network of the mRNAs and lncRNAs involved in the process. Here we use an RNA-Seq approach to comprehensively investigate temporal gene expression differences in skeletal muscle of rainbow trout after a single injection of E2. We also construct a functional lncRNA-mRNA regulatory network that is associated with skeletal muscle response to E2 treatment, and conduct a pathway enrichment analysis. This comprehensive analysis helps identify novel lncRNA and their related mechanisms with emphasis on regulation of muscle growth in rainbow trout under the influence of E2.

## Results

### Generation of a muscle transcriptome reference

To provide a comprehensive understanding of the effects of E2 treatment on muscle physiology, the lncRNA and mRNA transcriptomes were analyzed in skeletal muscle from E2-treated and control rainbow trout 24 and 72 hours post-injection. A total of 789,485,036 paired-end raw reads were generated from 16 samples with 101-bp read length (n = 4 per time and treatment combination). The number of sequences from each sample ranged from 36.2 to 63.8 million. After quality control including removal of ambiguous nucleotides, low-quality bases and ribosomal RNA sequences, a total of 749,490,934 cleaned reads (94.9%) were harvested for further analysis. The number of cleaned reads of each sample ranged from 34.5 to 60.9 million (Table 1).

**Table 1:** Summary of samples and RNA-Seq data. CTRL indicates control group. E2 represents estradiol treatment group.

Group	Time (h)	Replicate	Reads	Clean reads	Mapped reads	Mapping ratio (%)
CTRL	24	1	36,802,826	35,432,594	31,145,250	87.9
		2	61,291,464	52,228,282	46,274,258	88.6
		3	59,111,132	56,868,138	50,328,302	88.5
		4	50,601,976	48,340,122	42,974,368	88.9
	72	1	63,019,266	60,702,946	54,389,838	89.6
		2	60,452,644	58,114,660	52,186,986	89.8
		3	51,642,380	49,378,116	44,045,278	89.2
		4	63,821,958	60,981,886	53,786,022	88.2
E2	24	1	61,743,118	59,403,804	52,394,128	88.2
		2	37,513,122	35,786,668	30,186,188	84.3
		3	42,203,620	40,341,512	34,895,386	86.5
		4	47,655,922	45,563,586	40,232,838	88.3
	72	1	36,221,406	34,809,018	30,144,800	86.6
		2	41,421,664	39,634,688	34,878,528	88.0
		3	38,926,574	37,367,184	31,874,208	85.3
		4	37,055,964	34,537,730	29,586,296	85.6
Total			789,485,036	749,490,934	659,322,674	

The cleaned reads were pooled and assembled by Trinity^17^. Then CD-HIT-EST^18^ was used to remove the redundancy. As shown in Table 2, the transcriptomes were assembled into 243,509 contigs (203,148 Trinity components), ranging from 201 to 20,635 bp in length. To provide an expressed transcriptome reference and filter out transcripts that had very low read counts, EdgeR^19^ was used to remove the transcripts with count-per-million (CPM) less than 1. This produced 63,181 contigs (31,419 Trinity components), again ranging from 201 to 20,635 bp in length. The average length was 1,466 bp, N50 length was 1,982 bp and median length was 1,189 bp (Table 2). All cleaned reads were mapped to the expressed transcriptome reference by using the ultrafast short read aligner Bowtie^20^. The mapping ratio ranged from 84.3% to 89.8% with an average of 87.7% (Table 1).

**Table 2:** Statistics of transcriptome assembly.

Assembly	Number of components	203,148
	Number of contigs	243,509
	Maximum contig length	20,635 bp
	Minimum contig length	201 bp
	Average contig length	660 bp
	Median contig length	365 bp
	N50 length	1,076 bp
Filtered Assembly	Number of components	31,419
	Number of contigs	63,181
	Maximum contig length	20,635 bp
	Minimum contig length	201 bp
	Average contig length	1,466 bp
	Median contig length	1,189 bp
	N50 length	1,982 bp

### Identification of differentially expressed genes

**Figure 1:**
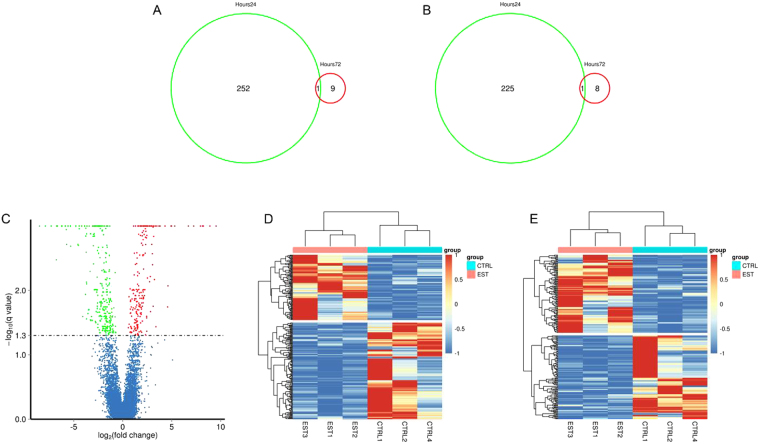
The number of differentially expressed genes in rainbow trout treated with estradiol (E2). (**A**) Venn diagram of common differentially expressed mRNA in E2 treated fish between 2 time points (24 hours post-injection versus 72 hours post-injection). (**B**) Venn diagram of common differentially expressed lncRNA in E2 treated fish between 2 time points. (**C**) A volcano plot of differentially expressed transcripts (lncRNAs and mRNAs) between control and E2 treated group. (**D**–**E**) Unsupervised hierarchical clustering of the expression profiles of differentially expressed mRNAs (**D**) and lncRNAs (**E**) both distinguish E2 treated and control group. Up- and down-regulated genes are indicated respectively by red and blue colors.

The pipeline reported in our previous study^21^ was used to identify all lncRNAs from the expressed transcriptome reference. Two biological replicates (CTRL3 & EST4) at 24 hour time point were identified as outliers based upon multidimensional distance scaling analysis and therefore were excluded. Finally, differential expression analyses were performed of coding genes and lncRNAs in E2 treated fish at 24 hours and 72 hours compared with the respective control fish. Given a false discovery rate (FDR) of 5%, 479 (226 lncRNA and 253 mRNA) differentially expressed genes (DEGs) were detected between E2 and control fish at 24 hours while only 19 DEGs (9 lncRNA and 10 mRNA) were detected at 72 hours (Supplementary Table S1 & S2). Only one lncRNA and one mRNA were differentially expressed at both time points (Fig. 1A,B). Due to the transient nature of the response, an extensive analysis of DEGs were completed only on differentially expressed lncRNA and mRNA detected 24 hour post-injection.

Transcriptome analysis identified 102 up-regulated and 151 down-regulated mRNAs and 119 up-regulated and 117 down-regulated lncRNAs in fish 24 hours post-E2 injection (Fig. 1C–E). Heat maps were generated using differentially expressed mRNAs (Fig. 1D) and lncRNAs (Fig. 1E) that self-segregated into control (CTRL) and EST (E2 treated) clusters in an unsupervised hierarchical clustering analysis. There were 36 genes that were differentially expressed more than 16-fold between control and E2 treated samples, of which 14 were up-regulated and 12 were down-regulated by E2.

### Experimental validation of lncRNA and mRNA

**Figure 2:**
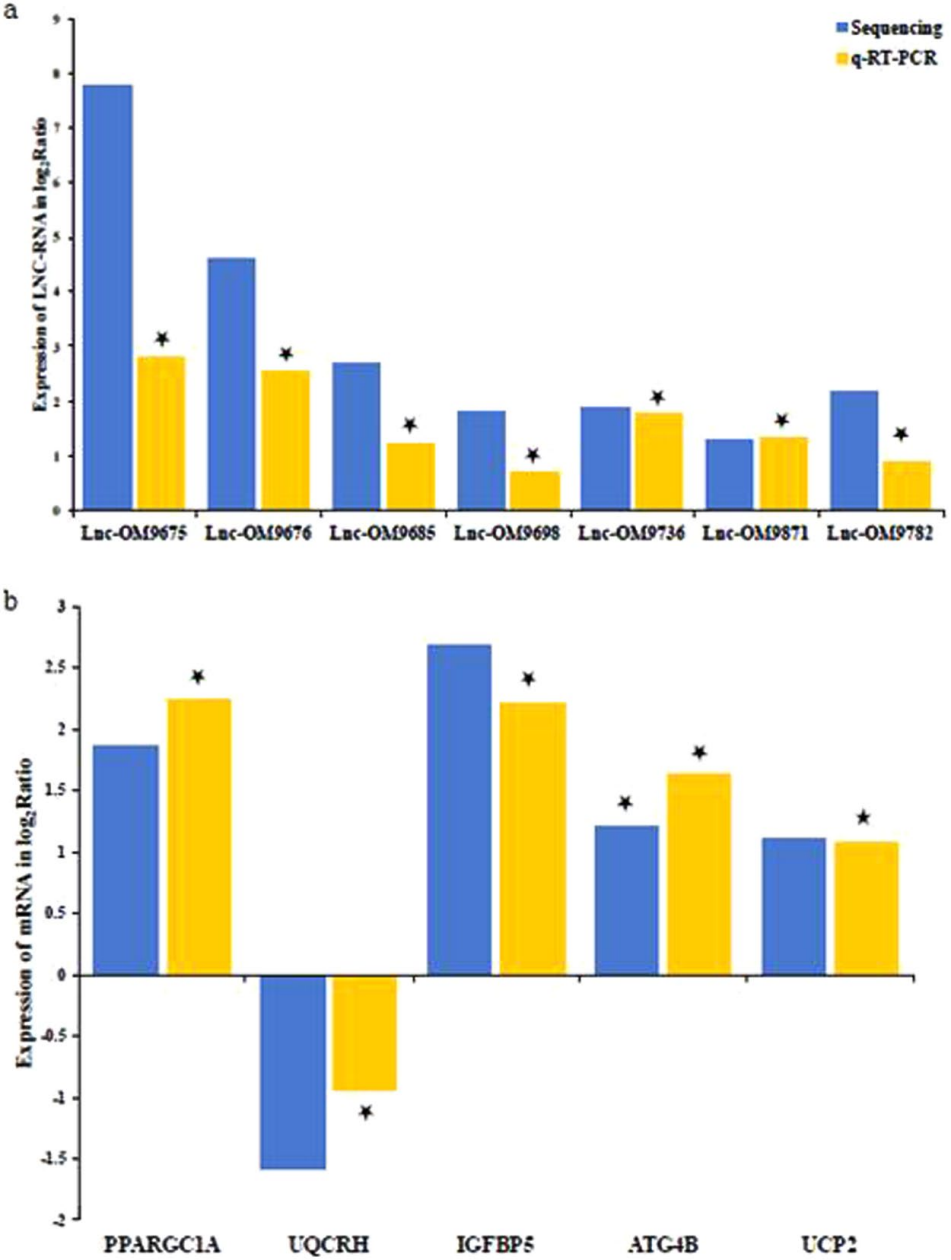
Differentially expressed lncRNAs and mRNAs validated by qRT-PCR. Comparison between RNA-seq and qRT-PCR validation results. X-axis shows lncRNAs (**A**) and mRNAs (**B**) validated in this study, relative expression normalized to β-actin; Y-axis shows log_2_Ratio of expression of E2 versus control. Stars indicate significant difference based on t-test (p < 0.05).

To confirm the sequencing results, seven lncRNAs and five mRNAs that were differentially expressed were randomly selected for validation using real time RT-PCR. Each transcript was confirmed as differentially expressed in E2-treated fish in the same direction as indicated by the RNAseq analysis (Fig. 2A,B).

### Differentially expressed genes represent several important pathways

**Figure 3:**
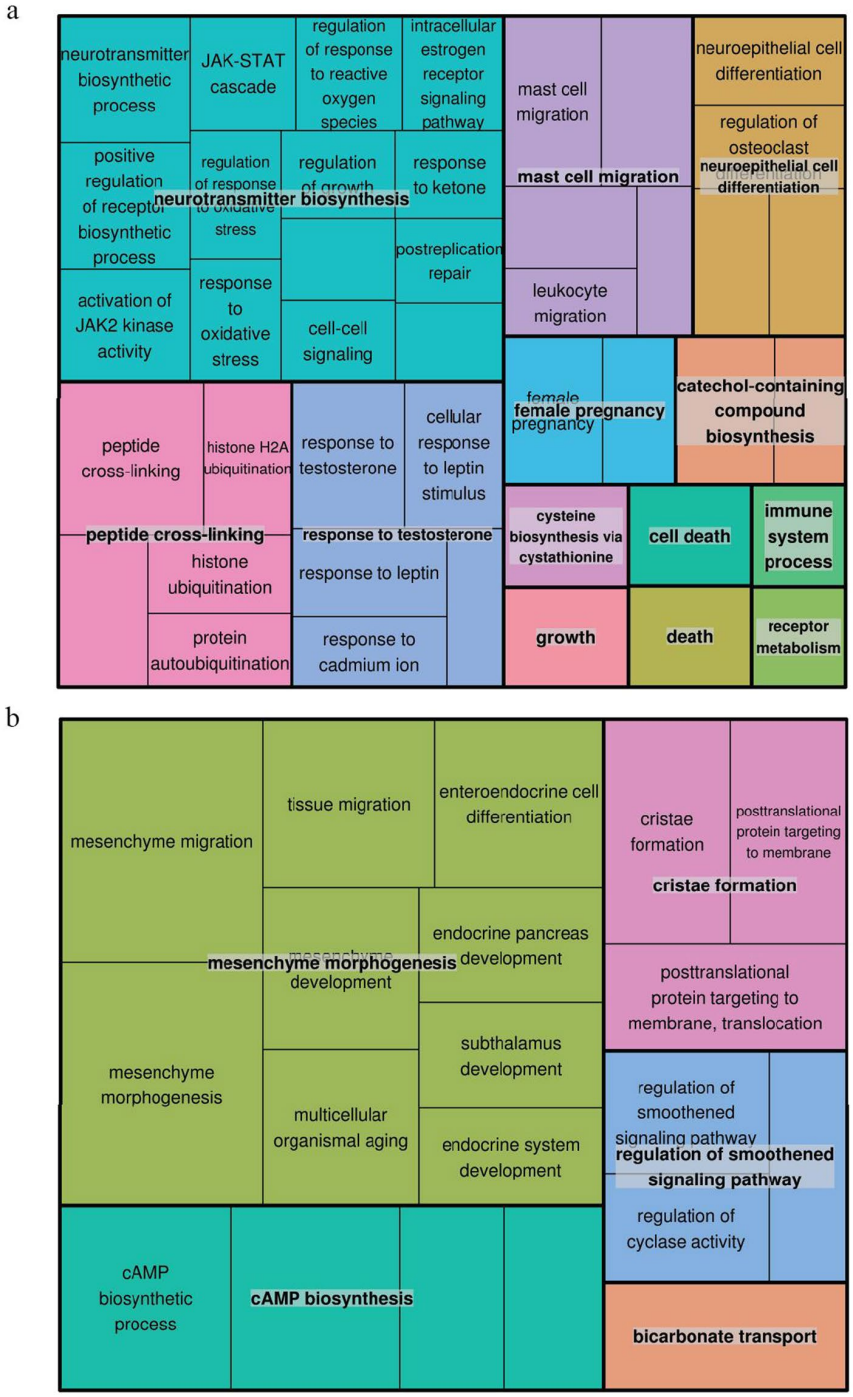
GO analysis of differentially expressed mRNAs. (**a**) Treemap of up-regulated representative GO terms in biological processes. (**b**) Treemap of down-regulated representative GO terms in biological processes. Each rectangle is a single cluster representative. The representative GO terms were joined into ‘superclusters’ of loosely related terms with same color.

To obtain the functions of the differentially expressed mRNAs and connections among them, we performed GO term and KEGG pathway enrichment analyses. To comprehensively interpret the GO term enrichment result, treemap was constructed based on the result of semantic similarity analysis. In each treemap, related terms were joined into a ‘supercluster’ with the same color and the most significant term as the representative of the group. As shown in Fig. 3a,b, in response to E2, skeletal muscle tissue showed notable up-regulations related to oxidative stress response, hormone response, protein ubiquitination, cysteine biosynthesis and DNA repair, and down-regulations mostly related to mesenchyme morphogenesis and cAMP biosynthesis in biological process. Notably, the term of response to leptin (GO:0044321) was identified to be enriched (Supplementary Fig. S1). Up-regulated genes in molecular function relate to kinase activity, hormone receptor activity, amino-acyl transferase activity and ubiquitin conjugating enzyme activity. Down regulated genes related to molecular function were related to muscle structural protein binding, motor activity, pyruvate carboxykinase activity and glucose-6-phosphate activity (Supplementary Fig. S2a,b). The enrichment of up-regulated genes in cellular component largely related to chromosome and ubiquitin conjugating enzyme complex, and down-regulated genes related to skeletal muscle structural protein complex (Supplementary Fig. S3a,b).

**Figure 4:**
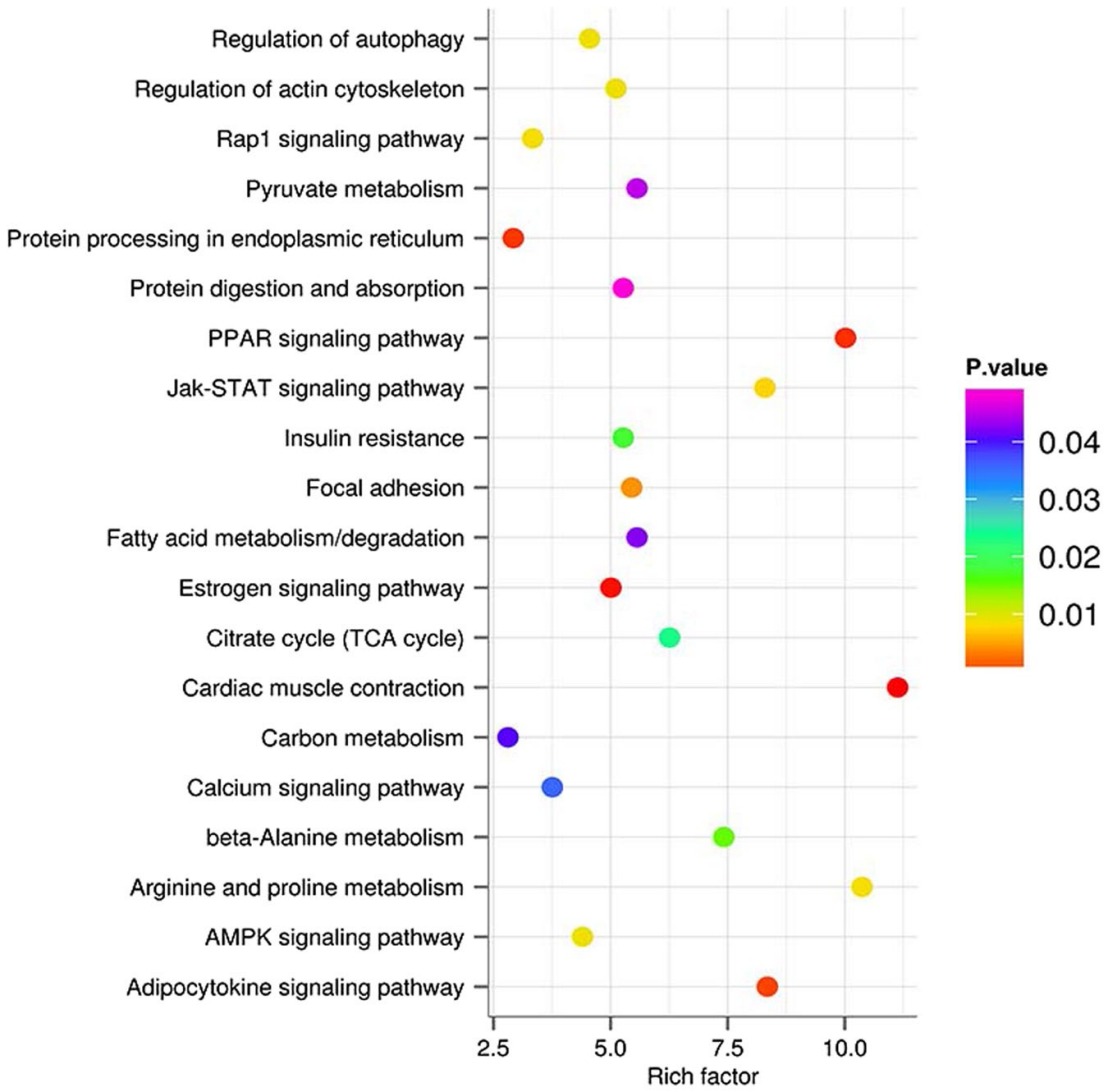
KEGG pathway analysis of differentially expressed genes. The rich factor was calculated using the gene count divided by the expected gene count.

The KEGG pathway analyses mapped the DEGs to KEGG reference pathways to infer systemic biological behaviors. Comparing to the control group, we observed significant KEGG pathway enrichment of DEGs in skeletal muscle in response to E2 stress. Of the top 20 over-represented KEGG pathways (Fig. 4), four were involved in signal transduction, including Rap1 signaling pathway, Jak-STAT signaling pathway, calcium signaling pathway and AMPK signaling pathway. An additional four pathways were associated with the endocrine system, which are PPAR signaling pathway, insulin resistance, estrogen signaling pathway and adipocytokine signaling pathway. Finally, the remaining 12 over-represented KEGG pathways were classified into different functional groups, including amino acid and carbohydrate metabolism, cellular community, protein synthesis and digestion.

### Construction of lncRNA-mRNA co-expression network

**Figure 5:**
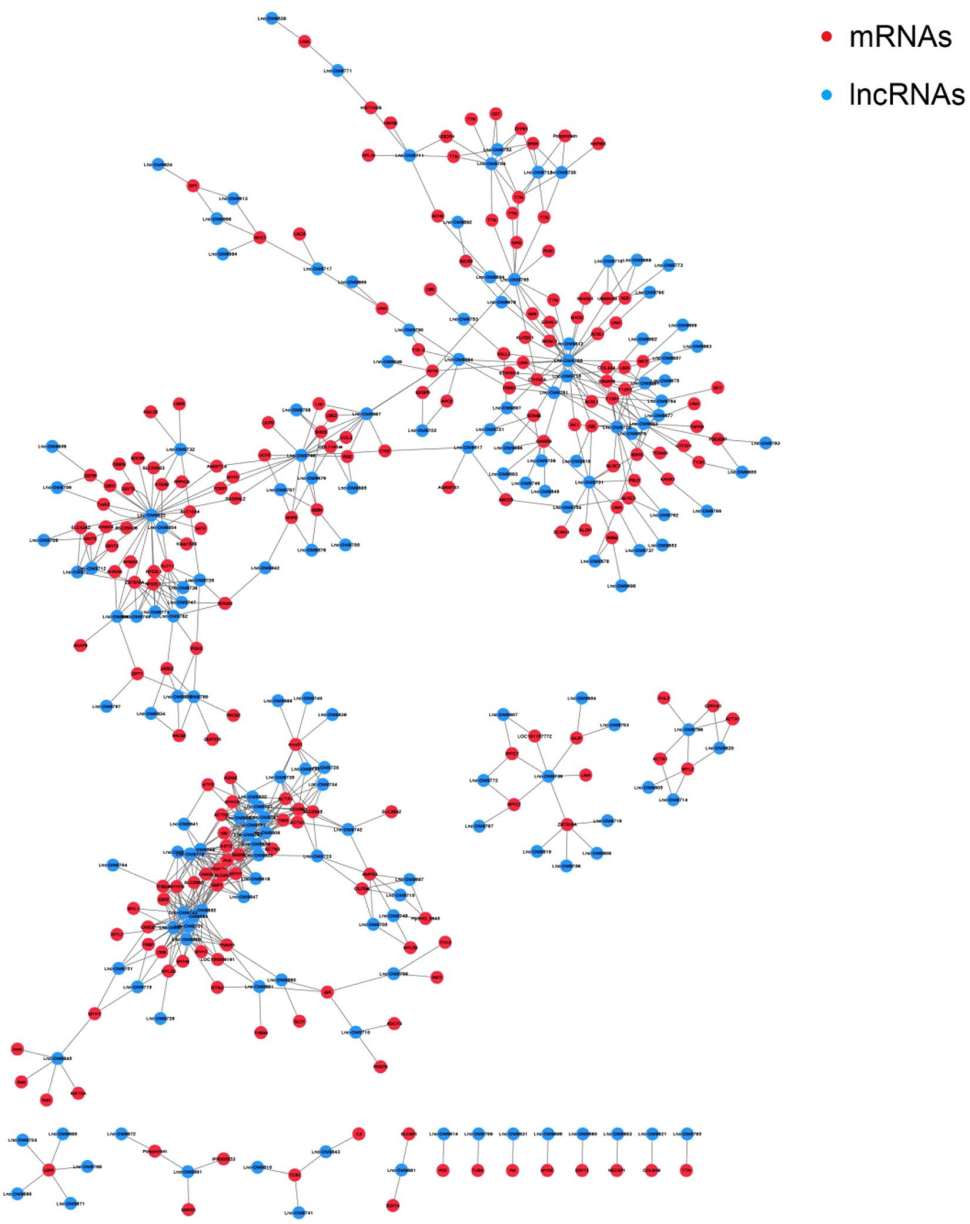
lncRNA-mRNA co-expression network. The differentially expressed lncRNA-mRNA co-expression network consisted of 681 co-expression relationship between 164 lncRNAs and 201 mRNAs. Blue circles represent lncRNAs, and red circles denote mRNAs.

To construct the differentially expressed lncRNA-mRNA co-expression network, the normalized expression values of the lncRNAs and mRNAs were obtained by using DESeq2^22^. Subsequently, Pearson’s correlation coefficient (PCC) was calculated between the normalized expression values of each of the lncRNA-mRNA pairs. The lncRNA-mRNA pairs with a PCC greater than 0.99 and FDR less than 0.05 were selected for network construction. The co-expression network consisted of 681 co-expression relationships between 164 lncRNAs and 201 mRNAs (Fig. 5). We then considered the node degree of the network, as a higher degree indicated that the nodes were likely to be hubs and therefore involved in more competing interactions. As a more stringent threshold, the top 5% (top 20) of the nodes were defined as hubs (Table 3). These 20 hub nodes that contain 14 lncRNAs co-expressed with more than 40% of the nodes in the network, implying the centrality of these nodes.

**Table 3:** Top 20 nodes with the highest degree in differential lncRNA-mRNA co-expression network.

Gene ID	Degree	Gene name	Symbol
TRINITY_DN61993_c9_g1	29	Lnc-OM9822	
TRINITY_DN89620_c0_g1	25	Lnc-OM9785	
TRINITY_DN61638_c0_g1	18	Lnc-OM9852	
TRINITY_DN62639_c0_g4	16	Lnc-OM9744	
TRINITY_DN60962_c3_g3	16	Lnc-OM9748	
TRINITY_DN57241_c1_g3	15	Lnc-OM9787	
TRINITY_DN38889_c0_g1	14	Lnc-OM9826	
TRINITY_DN33630_c0_g1	14	Lnc-OM9694	
TRINITY_DN57241_c2_g3	14	ADP/ATP translocase 2	ADT2
TRINITY_DN60357_c0_g5	13	Lnc-OM9743	
TRINITY_DN58760_c7_g3	13	Lnc-OM9759	
TRINITY_DN46607_c0_g1	13	Lnc-OM9725	
TRINITY_DN48565_c0_g1	13	Sestrin 2	SESN2
TRINITY_DN63539_c4_g1	12	Actin alpha 1	ACTA1
TRINITY_DN59565_c3_g3	12	Calsequestrin-2	CASQ2
TRINITY_DN62931_c4_g5	12	Lnc-OM9693	
TRINITY_DN32746_c0_g2	12	Lnc-OM9780	
TRINITY_DN62730_c1_g2	11	E3 ubiquitin-protein ligase	TRIM39
TRINITY_DN55042_c2_g2	11	Anion exchange protein 3	SLC4A3
TRINITY_DN60070_c2_g5	11	Lnc-OM9778	

**Figure 6:**
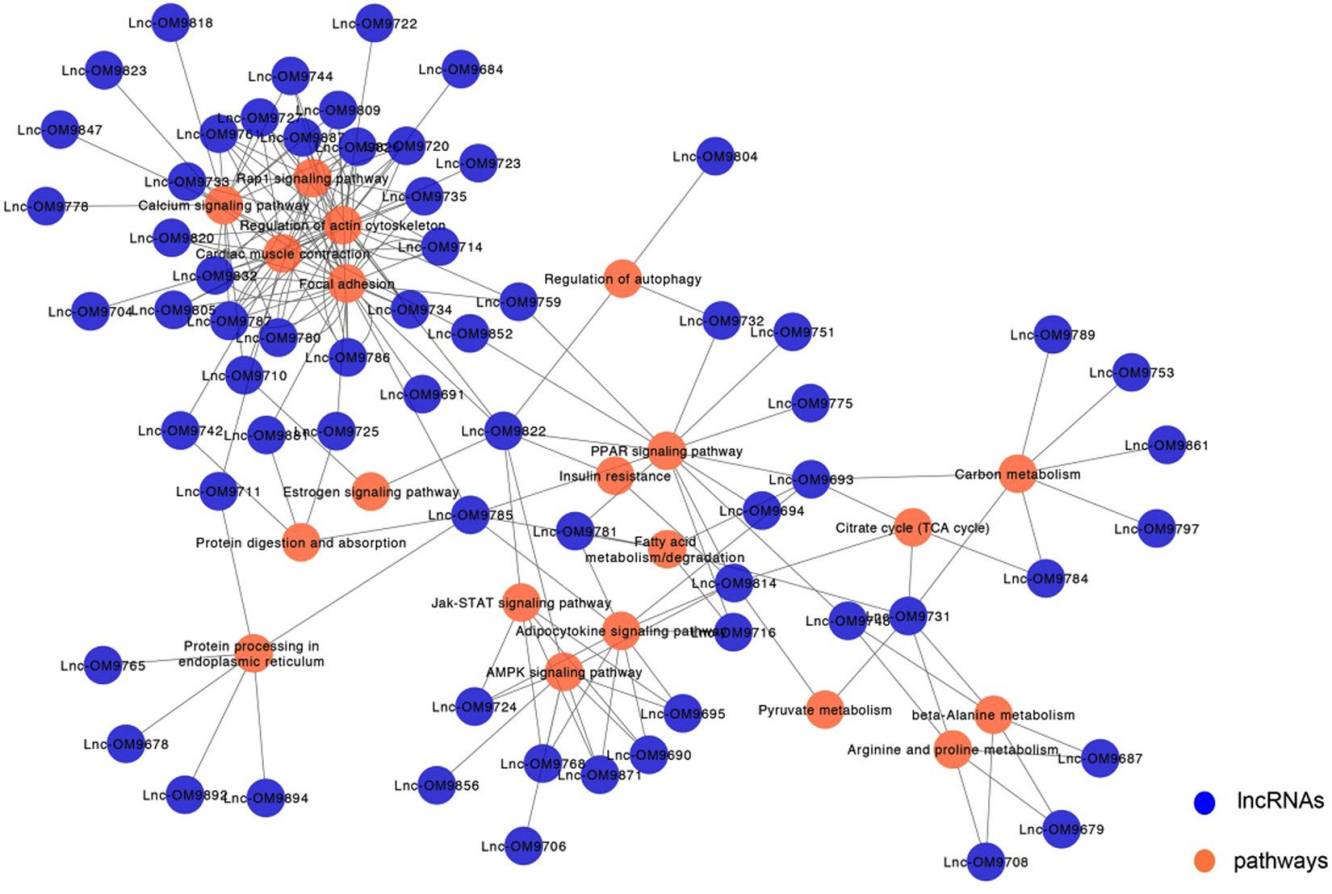
lncRNA-pathway network. Blue circles represent lncRNAs, and orange circles denote pathways.

A lncRNA-pathway network was constructed to identify the possible functions of lncRNAs, in which nodes represent lncRNAs or over-represented pathways. Connections between nodes were made if mRNAs that co-express with a lncRNA associate with a common pathway, thus supporting that the pathway is regulated by the corresponding lncRNAs (Fig. 6). Sixty-five lncRNAs (Supplementary Table S3) were linked with 20 enriched pathways (Table 4, Supplementary Table S4) in the lncRNA-pathway network, suggesting these lncRNAs play a central role in regulating the E2 response.

**Table 4:** Key pathways in E2 treated skeletal muscle.

Regulatory lncRNAs (n)	Pathway
30	Focal adhesion
28	Regulation of actin cytoskeleton
17	Cardiac muscle contraction
15	Calcium signaling pathway
15	Rap1 signaling pathway
13	PPAR signaling pathway
10	Adipocytokine signaling pathway
9	AMPK signaling pathway
7	Carbon metabolism
6	Jak-STAT signaling pathway
6	Protein processing in endoplasmic reticulum
5	Arginine and proline metabolism
5	Fatty acid metabolism/degradation
5	Beta-Alanine metabolism
4	Citrate cycle (TCA cycle)
4	Protein digestion and absorption
3	Regulation of autophagy
2	Estrogen signaling pathway
2	Insulin resistance
2	Pyruvate metabolism

## Discussion

In rainbow trout, E2 is a maturation-related signal that negatively affects muscle growth. The present study identified lncRNAs and mRNAs regulated by E2 in rainbow trout skeletal muscle, thereby identifying mechanisms contributing to E2-induced muscle catabolism. Association analysis suggests that lncRNAs are central regulators of multiple components within a single pathway, supporting lncRNAs as important mechanisms for muscle physiology and the E2 response.

LncRNAs are emerging as regulators of diverse biological functions^23^. We identified 226 lncRNAs that were differentially expressed, suggesting that lncRNAs are regulated by E2 and likely contribute to the catabolic effects of this steroid in skeletal muscle. Only three of the differentially expressed lncRNAs (lnc-OM206, 3272 & 7282) were previously reported in a rainbow trout lncRNA reference transcriptome pooled from 15 tissues, including muscle^21^. This lack of overlap suggests that lncRNAs are temporospatially expressed, introducing the need for greater coverage of the rainbow trout lncRNA transcriptome across various tissues, periods of development, and treatment conditions. In particular, the sensitivity of lncRNAs (and mRNAs) to treatment conditions are supported by this study with the dramatic reduction in number of DEGs between 24 and 72 hr post-injection.

The functional roles of lncRNAs are poorly characterized. Co-expression models assist in large-scale prediction of lncRNA functions through integrating expression profiles of protein-coding genes and lncRNAs (coding-non-coding gene co-expression network)^24,25^. In this study a similar co-expression analysis was used to predict lncRNA functions based on their association with co-expressed genes, based on the fact that genes with similar expression patterns have a greater tendency to be involved in the same pathways^26^. The resultant co-expression network contained a total of 164 differentially expressed lncRNAs and 201 differentially expressed mRNAs. This network provides a global view of possible lncRNA-coding gene expression associations that are influenced by E2 treatment in salmonids. In this network, the top 5% (20) large degree nodes were all lncRNAs, of which, 14 lncRNAs co-expressed with more than 40% of the nodes. These observations suggest that lncRNAs have functional significance in the E2-induced reduction of muscle growth in rainbow trout.

To examine the key lncRNAs and their potential functions, lncRNA-pathway network was constructed based on pathway enrichment analysis. The lncRNA-pathway network contains 65 lncRNAs linked with 20 significantly enriched pathways. The top pathway containing the most lncRNAs linked was focal adhesion (FA). Focal adhesions are integrin-containing, multi-protein structures that mediate the regulatory effects of a cell in response to extracellular matrix (ECM) adhesion^27^ which also contribute to ECM degradation^28^. Our observation that E2 induces regulation of lncRNAs associating with FAs may contribute to changes in the structure and functional capacity of the skeletal muscle ECM. In mammals skeletal muscle ECM adapts to conditions that affect muscle growth such as injury or disease^29^, conditions that produce similar negative effects on muscle growth induced by E2 in salmonids^8,30^. Additionally, spawning female rainbow trout exhibiting muscle atrophy display reduced expression of ECM and FA-related genes^31^, supporting that regulation of related proteins represents muscle restructuring.

In addition to FA, we also found that calcium signaling pathway was linked with 15 lncRNAs in our lncRNA-pathway network. Calcium ions are important for cellular signaling, which plays a pivotal role in almost all cellular processes. Calpain is a protein belonging to the family of non-lysosomal cysteine proteases that is activated by calcium ions. Calpains function in various biological processes in skeletal muscle, including apoptosis^32^, proteolysis of myofibrillar proteins^33^, and myogenic events^34^. Increased caplain expression occurs concurrently with elevated E2 during spawning in rainbow trout^35^, however, regulation of calpastatins, the calpain inhibitors, also occurs during this period^36^, which is under direct effect of E2^12^. Therefore, regulation of lncRNAs affecting calcium signaling capacity is likely significant for E2-induced elevations in muscle protein degradation. Probably further affecting calcium signaling was down-regulation of calsequestrin, a calcium-binding protein that regulates calcium homeostasis^37^. Our previous findings suggest that target genes of miR-23a-3p are involved in mitochondrial outer membrane permeability^11^; calcium signaling also plays a vital role in mitochondrial permeability which results in cell death. Additional lncRNAs regulated by E2 in skeletal muscle have functional roles in nutrient metabolism, including amino acids, fatty acids, and pyruvate, as well as protein degradation. As E2 shifts nutrient partitioning from muscle accretion in support of gonad development, the energy requirements of muscle are expected to change. These data support a role for lncRNA as regulators of these processes. Although the results of the present study require further experimental verification, they provide insight into the significant role that lncRNAs have in the physiological and biochemical response to E2 in skeletal muscle of rainbow trout.

Various genes functioning as molecular factors, cellular components and those involved in biological processes were up-regulated in rainbow trout skeletal muscle under the influence of E2. These proteins function to regulate different signaling pathways including JAK/STAT pathway, response to oxidative stress, cell-cell signaling and intracellular estrogen receptor signaling pathways with positive regulation of various receptor biosynthesis and their binding activity (insulin receptor binding, insulin like growth factor binding, integrin binding etc). Besides, important protein cross-linking molecules involved in histone ubiquitination, protein autoubiquitination, and their responsible enzymes like ubiquitin conjugating enzyme and its complex were also up-regulated. Elevated expression of important metabolic enzymes, including aldehyde dehydrogenase and succinate dehydrogenase, important for energy synthesis was observed.

Estradiol induces the activation of estrogen receptors that are functional through genomic and non-genomic pathways^38^. Besides activation of estrogen receptors, E2 is recognized as a regulator of the IGF pathway in salmonids^39^. IGFBP-5, an IGF binding protein, was reported to be highly conserved among humans, rats (*Rattus norvegicus*), mice and frog (*Xenpus laevis*), and is abundantly expressed in skeletal muscle during myoblast differentiation in mouse^40^. This response inhibits muscle cell differentiation by binding to the IGF receptor and impeding IGF activity^41^. In the current study, real time PCR and RNAseq analysis both support increased IFGBP-5 expression after 24 hours of E2 treatment, and a similar response was observed in a previous rainbow trout E2 injection study^14^. However, long-term E2 exposure (30 days) in rainbow trout had the opposite effect^8^. The differential time-dependent response could reflect the complex and dynamic nature of the IGF endocrine system, in which relative concentrations of both IGF and IGF binding proteins regulate IGF binding to receptors. Collectively these observations support that regulation of IGFBP-5 in muscle is important for effects of E2 on muscle cell differentiation.

Production of reactive oxygen species leading to oxidative stress as a response to E2 was reported in Japanese medaka (*Oryzias latipes*) and in Japanese sea bass (*Lateolabrax japonicus*)^42–44^. DNA damage induced by reactive oxygen species was reported in fathead minnows (*Pimephales promelas*) and Japanese sea bass exposed to E2^44,45^. Reactive oxygen species (ROS) contribute to autophagy^46^ and muscle atrophy by activating muscle degrading pathways involving calpains, caspase-3, ATG4b, MuRF-1 and atrogin-1^47^. Supporting this concept is our previous report of increased expression of caspase-9, caspase-3 and atrogin-1 by E2 in rainbow trout^11^. *In vivo* and *in vitro* experiments in rainbow trout and rainbow trout primary myocytes showed that exposure to E2 increased expression of atrogin-1/fbxo32 and MURF genes and increased protein degradation^11,48^. Atrogin-1 is a ubiquitin ligase responsible for ubiquitinating specific proteins such as MyoD and myogenin for proteasomal degradation^49,50^, and increased atrogin-1 expression is typically associated with catabolic conditions in both fish and mammals^51,52^. Supporting E2-induced reductions in muscle protein retention in rainbow trout was increased expression of autophagy related 4b cysteine peptidase (ATG4b) and previous literature reports a similar response for additional components of the autophagy-lysosome system^8,12^. Collectively, these findings indicate the regulation of E2 on the skeletal myogenic program and mechanisms regulating protein retention and muscle accretion.

Characterized GO terms that showed reduced expression in E2-treated skeletal muscle after 24 hours are mainly involved in structural make up of skeletal muscle. Significantly down regulated proteins are primarily involved in actin cytoskeleton, actin binding, motor activity and mesenchymal morphogenesis. Additionally, GO terms related to cAMP biosynthesis, cristae formation, regulation of smoothened signaling pathway, and RNA mediated DNA polymerase activity were differentially regulated. In humans and mouse mesenchymal stromal cells are multipotent cells that differentiate to different cell lineages including mesodermal cells considered as precursors for myoblasts or MPCs^53,54^. The bone marrow derived mesenchymal cells with myogenic markers migrate to sites of muscle regeneration^55,56^. In the current study, the observed decrease in the genes responsible for mesenchymal cell migration and morphogenesis may have negative effects on muscle development. Wnt signaling in rat is necessary for differentiation of mesenchymal cells to myogenic origin^57^, which also inhibits their differentiation to adipogenic origin by decreasing the expression of CCAAT enhancer binding protein alpha and peroxisome proliferator-activated receptor gamma (PPARγ)^57^. Peroxisome proliferator-activated receptor gamma coactivator 1 alpha (PPARGC1A) is a coactivator of PPARγ and highly expressed in mouse skeletal muscle^58,59^. Studies also indicate that PPARGC1A and PPARGC1B play prominent roles in estrogen receptor signaling pathway by acting as cofactors to enhance transactivation of estrogen receptor α^60–62^. In our study, PPARGC1A exhibited increased expression in skeletal muscle within 24 hours of E2 exposure, supporting that effects of E2 in muscle extend to regulating mechanisms affecting mesenchymal cell differentiation and fiber type.

In summary, our comprehensive analyses provide novel knowledge of mRNAs and lncRNAs at the transcriptomic level regarding the influence of E2 on rainbow trout skeletal muscle within a relatively short period after steroid injection (24 hr). These results and conclusions will serve as important resources for future experiments that further investigate the role and regulation of lncRNAs in rainbow trout. The dynamic expression response observed in the current study may be driven by a dramatic reduction in plasma E2 between 24 and 72 hrs post-injection due to high E2 clearance. A previous study using an identical injection approach reported a greater than 3-fold reduction in plasma E2 between these sampling periods (121 ng/ml vs 36 ng/ml)^14^. Therefore, an experimental model that enables sustained elevated plasma E2 would be especially valuable to mimic the steady-state elevation in E2 production during sexual maturation and further coordinate the gene expression data with changes in body weight and dynamics of muscle accretion.

## Methods

### Ethics statement

All animal experiments in this study were performed at the USDA/ARS National Center for Cool and Cold Water Aquaculture (NCCCWA) and approved by the NCCCWA Institutional Animal Care and Use Committee (protocol #50).

### Experimental design

A total of forty fish weighing approximately 40 g were randomly assigned to four experimental tanks (n = 10 fish per tank). The study consisted of two treatments, including intraperitoneal injections of E2 and the delivery vehicle to serve as the control. Treatments were applied to fish in duplicate tanks. Estradiol was resuspended (10 μg/μL) in 95% ethanol and diluted to 2.5 μg/μL with vegetable oil. The control treatment contained an equal ratio of ethanol: vegetable oil as compared to E2 suspension. Estradiol and the vehicle injection methodology was adapted from previously published procedures used in tilapia^63^. Feed was withheld the day of E2 injection and throughout the study period. Fish were anesthetized with tricaine methanesulphonate (MS-222, 100 mg/l), weighed, and received intraperitoneal injections (2.0 μl/g body weight) of E2 (5.0 μg/g body weight) or the vehicle. Fish in one tank per treatment were each harvested 24 and 72 hours post-injection using lethal dose of MS-222 (300 mg/l). Skeletal muscle was dissected and immediately frozen using liquid nitrogen for further processing.

### RNA extraction and quality control

Total RNA was extracted from skeletal muscle samples of fish treated with E2 and controls at 24 and 72 hours (6 biological replicates each) using TRIZOL reagent (Invitrogen, Carlsbad, CA) per the manufacturer’s suggested protocol. Quality and quantity of RNA was estimated using the A_260_:A_280_ ratio. Integrity and size distribution of RNA was evaluated using a Bioanalyzer 2100 (Agilent technologies, Santa Clara, CA). Four replicates of each treatment were subject to RNAseq.

### cDNA library construction and sequencing

Library construction (poly(A)-enriched) and sequencing was completed by the Johns Hopkins University Genetic Resources Core facility High Throughput Sequencing Center (Baltimore, MD). cDNA libraries were constructed using TruSeq RNA library preparations and high throughput sequencing was completed using the Illumina HiSeq platform (100 bp single reads).

### Sequence data processing, de novo assembly and differential expression analysis

Adaptor sequences were trimmed and ambiguous and low quality bases were removed, then read lengths less than 50 were removed. TRINITY^17^ was used to assemble all cleaned reads with default parameters. CD-HIT-EST was used to remove the shorter redundant transcripts when they were 100% covered by other transcripts with more than 99% identity^18^. All the cleaned reads were mapped to the assembled transcriptome by Bowtie^20^. RSEM was used to estimate and quantify gene expression levels from RNA-Seq data^64^. The final counts matrix file was used as input for the R package edgeR^19^ The exact test in edgeR was completed to discover the DEGs between the treatment groups. False discovery rate was used for multiple test correction. Any genes with a fold change greater than 2.0 and FDR of less than 0.05 were defined as DEG.

### Validation of sequencing results

Sequencing results were validated using real time RT-PCR. RNA from 24 hour samples (6 replicates from each treatment) was used for cDNA synthesis using miScript II (Qiagen, Valencia, CA). Expression of mRNA and lncRNA was normalized to β-actin, which was not identified as a DEG by RNAseq analysis. Real time RT-PCR followed by melt curve analysis was performed to determine the specificity of the amplicons. Standard curves with 10-time serial dilutions of a pooled cDNA sample were generated to calculate the efficiency of qPCR. Five μl of diluted cDNA (1:1024) was used with iQ™ SYBR® Green Supermix (Bio-Rad) and 300 nM of forward and reverse primer in a final volume of 25 μl for the reaction. Cycle conditions were the same for all the primers except the annealing temperatures for different primers. Initial denaturation at 95 °C for 3 minutes followed by a denaturation at 95 °C for 40 sec, annealing for 30 sec, extension at 72 °C for 30 seconds for 40 cycles and a final extension for 10 min at 72 °C. Melt curve analysis was performed with an increase of 0.5 °C increase every cycle. Quantification cycle (Cq) values were used for quantification of expression using the log-linear equation of standard curve. Relative fold changes were calculated by setting the values of the controls to 1.0 and comparing the respective treatment groups. Statistical analysis was performed using Student’s t-test and those with a *P*-value < 0.05 were considered statistically significant.

### LncRNA identification and GO and KEGG enrichment analysis of mRNAs

All DEGs were mapped to the rainbow trout genome^65^ using BLAT^66^. We used the pipeline reported in our previous study^21^ for lncRNA identification to detect differentially expressed lncRNAs. All differentially expressed mRNAs were subjected to similarity search against NCBI non-redundant (nr) protein database using BLASTx^67^ with an e-value cutoff of 1e-10. Gene names and GI were assigned to each mRNA based on the BLASTx result. ID mapping was performed using our in-house script to extract all associated GO terms for each mRNA. KEGG pathways were assigned to each mRNA using the online KEGG Automatic Annotation Server (KASS)^68^. The R package GOstats was used to run hypergeometric testing on GO and KEGG terms^69^. Redundant GO terms were removed by REVIGO, a Web server that summarizes long, unintelligible lists of GO terms by finding a representative subset of terms using semantic similarity measurement based clustering algorithm^70^.

### Co-expression analysis

To identify co-expressed lncRNA-mRNA pairs, Pearson’s correlation coefficients were calculated based on the normalized expression value between every differentially expressed lncRNA and mRNA pair. Only the strong correlations (≥0.99) were selected to construct the network. The threshold of FDR was < 0.05. Cytoscape was used to construct the co-expression network^71^.

### Data availability

The datasets generated during and/or analyzed during the current study are available from the corresponding author on reasonable request.

## Electronic supplementary material

Supplementary Figures S1-S3

Supplementary Table S1

Supplementary Table S2

Supplementary Table S3

Supplementary Table S4
