# Supplementary material for: Comprehensive analysis of lncRNAs and mRNAs in skeletal muscle of rainbow trout (Oncorhynchus mykiss) exposed to estradiol
Source: Sci Rep. 2017 Sep 18;7:11780. doi: 10.1038/s41598-017-12136-6 (PMC5603547; doi:10.1038/s41598-017-12136-6)
Supplement: Supplementary file 1 — Supplementary Figures S1-S3 [file 41598_2017_12136_MOESM1_ESM.pdf]

Manuscript Title: Comprehensive analysis of lncRNAs and mRNAs in skeletal muscle of rainbow trout (Oncorhynchus mykiss) exposed to estradiol
Authors: Jian Wang, Prasanthi P. Koganti, Jianbo Yao, Shuo Wei and Beth Cleveland

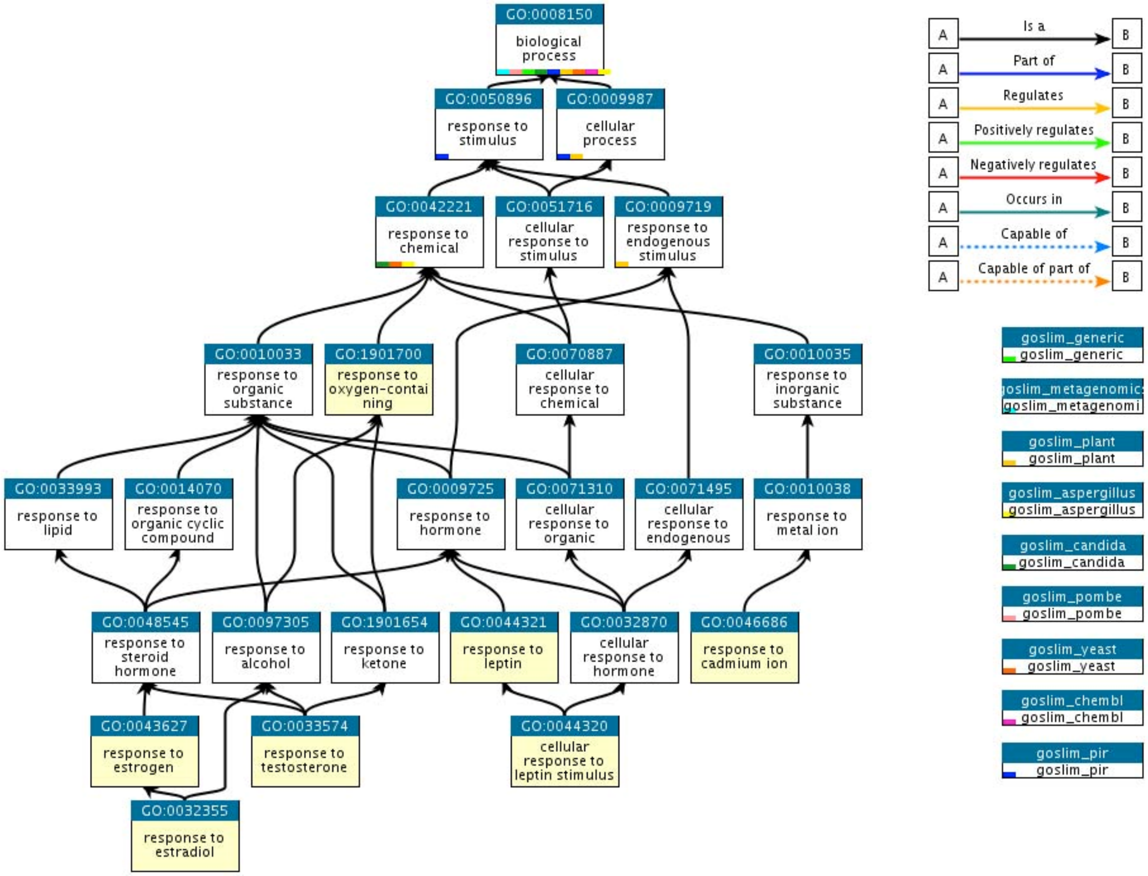

Supplemental Figure S1. Directed acyclic graph (DAG) of over-represented and hormone response associated GO terms.

Manuscript Title: Comprehensive analysis of lncRNAs and mRNAs in skeletal muscle of rainbow trout (Oncorhynchus mykiss) exposed to estradiol
Authors: Jian Wang, Prasanthi P. Koganti, Jianbo Yao, Shuo Wei and Beth Cleveland

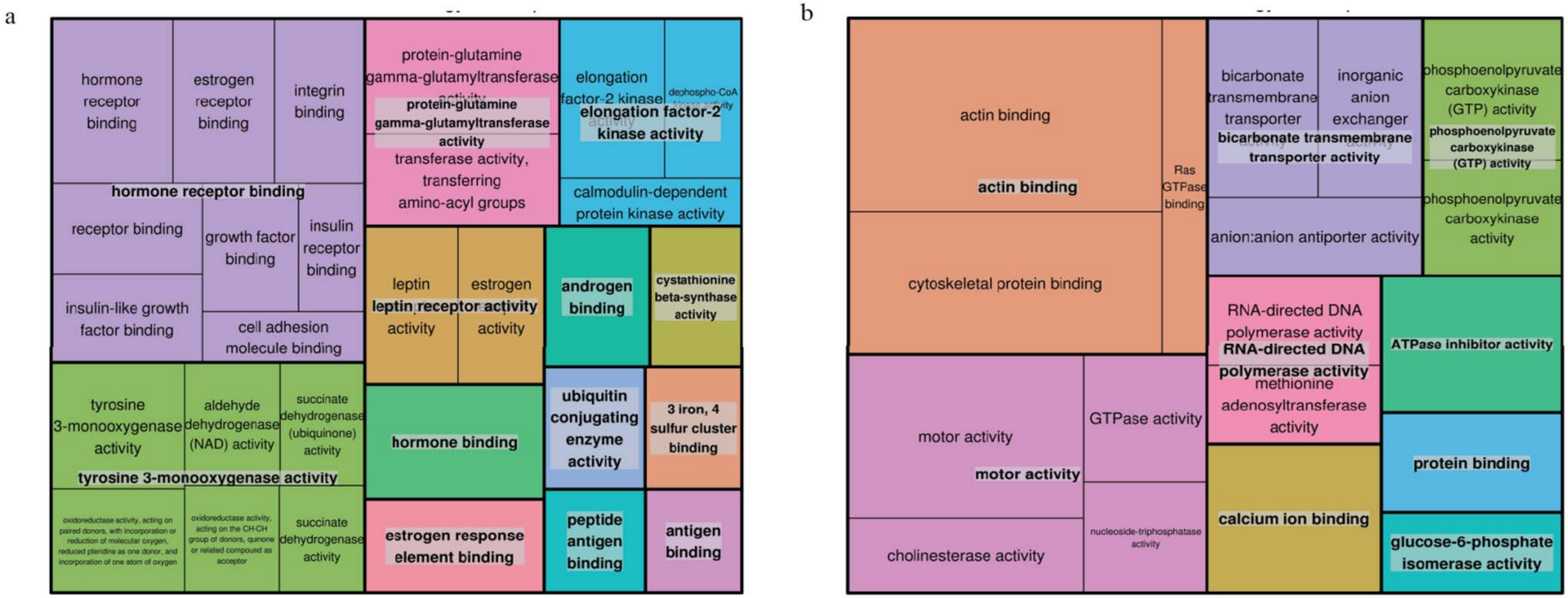

Supplemental Figure S2. GO analysis of differentially expressed mRNAs.
(a) Treemap of up-regulated representative GO terms in MF. (b) Treemap of down-regulated representative GO terms in MF. MF represents molecular function.

Manuscript Title: Comprehensive analysis of lncRNAs and mRNAs in skeletal muscle of rainbow trout (Oncorhynchus mykiss) exposed to estradiol

Authors: Jian Wang, Prasanthi P. Koganti, Jianbo Yao, Shuo Wei and Beth Cleveland

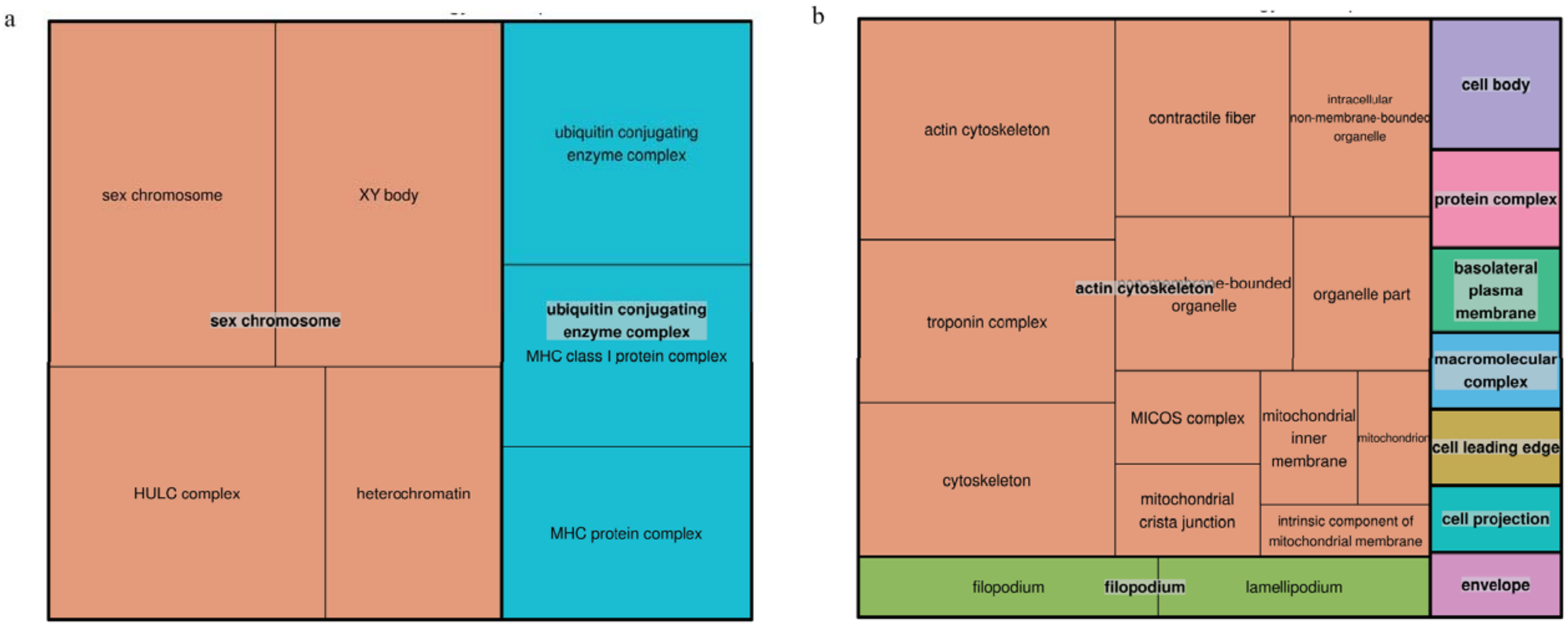

Supplemental Figure S3. GO analysis of differentially expressed mRNAs.
(a) Treemap of up-regulated representative GO terms in CC. **(b)** Treemap of down-regulated representative GO terms in CC. CC indicates cellular component.
